# Multimodal preoperative imaging for transcatheter mitral valve replacement in the domestic sheep model

**DOI:** 10.1038/s41598-024-62646-3

**Published:** 2024-05-23

**Authors:** John P. Carney, Richard W. Bianco

**Affiliations:** Experimental Surgical Services Laboratory, Department of Surgery, University of Minnesota Minneapolis, 425 East River Parkway KE B18, Minneapolis, MN 55455 USA

**Keywords:** Computed tomography, Echocardiography, Mitral valve, Transcatheter, Sheep model, Preclinical, Anatomy, Medical research, Preclinical research, Translational research

## Abstract

Preclinical in vivo evaluation is an essential step in the progression of new cardiac devices into patient use, with studies predominantly performed in the domestic sheep model. A growing area of interest in cardiac device development is transcatheter mitral valve replacement (TMVR). Clinically, multimodal imaging, or computed tomography (CT) and echocardiography (echo) are used extensively to preoperatively determine mitral valve morphology prior to an intervention, but there is no description on how these modalities can be implemented to support preclinical studies. The purpose of this study is to apply clinically relevant CT and echo acquisition and assessment techniques to a large group of naive research sheep in order to analyze and report modality-related effects on mitral valve dimensional reference intervals in the sheep model. To this end, fifty-five adult domestic sheep underwent preoperative CT and echo exams and resultant images were analyzed using a landmark-based multiplanar measurement protocol and compiled into a master dataset for statistical analysis. We found moderate agreement between CT and echo-derived measurements of the mitral valve in sheep and propose the first clinically-relevant dimensional indices for the sheep’s naive mitral valve which can be used to guide future studies evaluating novel TMVR devices. This study is the first of its kind in proposing a reproducible method for detailed examination of the mitral valve in the sheep model using clinically-relevant multimodal imaging. As in patients, CT and echo can reveal accurate native mitral valve dimensions in the sheep prior to preclinical TMVR studies.

## Introduction

The development of catheter-based valvular replacement devices has resulted in an ever-expanding array of options available for clinicians to treat structural cardiac valvular disease^1–6^. Traditional surgical valve replacement procedures require invasive surgical approaches for direct cardiac visualization reliant on cardiopulmonary bypass (CPB), while catheter-based devices can be inserted using minimally invasive interventional techniques. With the patient sedated and anatomy visualized by fluoroscopy and echocardiography (echo), an appropriately sized catheter-based device can be inserted through a peripheral vascular access site, advanced into the heart, and deployed within the diseased valve, drastically decreasing anesthesia time during the case, as well as reducing the postoperative recovery period.

Both echo and Computed Tomography (CT) have played a central role in preoperative planning for catheter-based valve replacement procedures^7^. Prior to a procedure, the heart team must comprehensively assess the cardiac disease and anatomy of the patient to select the appropriate catheter-based device and size for the intervention to follow. With complementary strengths and differing weaknesses, use of preoperative echocardiography and CT can provide the heart team with high fidelity images and understanding of the valvular disease to be treated prior to a catheter-based valve replacement^8–11^. As a result, this preoperative multimodal imaging strategy has found broad clinical use, most frequently in the treatment of aortic valve disease.

With current advancements in catheter-based valve replacement technology, there has been a dramatic shift in the treatment of aortic valve disease^2–6^. Transcatheter aortic valve replacement, or TAVR, is the most commonly used catheter-based valve replacement performed in adult cardiac patients to date^12,13^. Initially performed in high-risk patients with end stage heart failure, advancements in catheter technology, imaging, operator experience and postoperative outcomes has propelled this approach to a broader segment of the adult cardiac patient population—younger and healthier patients with symptomatic severe aortic valvular disease^12^. Prior to the advent and accessibility of TAVR, these patients would have been treated with surgical aortic valve replacement facilitated by cardiopulmonary bypass (CPB). Initial results from a multitude of clinical studies examining the efficacy and outcomes of TAVR appear to suggest that TAVR is associated with comparable if not improved postoperative outcomes as surgical aortic valve replacement^2–6,12,13^.

Building off the clinical success of TAVR, many investigators in the field are turning their attention to transcatheter mitral valve replacement, or TMVR. Mitral valve disease is the most common valvular disease in the adult population, with patient age being the primary driving factor of the disease^14,15^. While more common than aortic valve diseases, the current standard of care for treatment of mitral disease is either surgical valve repair or replacement, both requiring deep anesthesia and open heart surgery. As a result, patients suffering from mitral valve disease are treated an estimated 50% of the time, with surgical and anesthesia risk being the primary reason for treatment exclusion^15^. A minimally-invasive, catheter-based device suitable for the mitral valve has great potential for treating high-risk patients with few current options. Like in TAVR, TMVR could be another disruptive improvement to the standard of care for patients suffering from mitral valve disease.

Currently, regulatory agencies require robust and comprehensive feasibility and safety evaluations of novel cardiac devices in large animal models prior to clinical study and use^16–19^. One of the most commonly used animal models for such preclinical studies is the domestic sheep (Ovis aries). Use of the sheep model in evaluating novel cardiac devices and their translation to the clinical setting has been widely reported in published literature, especially in evaluating long-term hemodynamic performance and durability, thromboembolic complications, and biocompatibility^16–24^. As a result, the sheep has become the standard large animal model for in vivo testing and evaluation of novel cardiac valve replacement devices prior to human use^21^.

Citing the widespread use of preoperative multimodal imaging in the forms of echocardiography and CT scanning, investigators developing novel TMVR devices are looking to apply these clinical strategies to their preclinical animal studies. Investigators are hoping to leverage this preoperative imaging to make their in vivo study more relevant and translatable to clinical studies while improving preclinical study outcomes, especially for outcomes driven by appropriate cardiac sizing and animal selection for a specific TMVR device design. However, no information exists in the literature as to cardiac anatomical dimensions in sheep as imaged by CT or echo, nor examples of applying clinical methodologies to the preoperative evaluation of the mitral valve prior to a TMVR procedure. Therefore, the objectives of the present study were to (1) investigate the feasibility and reproducibility of measuring mitral annular dimensions with clinically used CT reconstruction techniques, (2) characterize the dimensions of mitral valve annular and associated heart structure dimensions, and (3) compare CT-derived measurements to measurements collected from echo exams performed in the same population to test the effect of imaging modality on cardiac valve sizing. In completing this analysis, we are proposing the first reproducible method for a structural assessment of the sheep model’s native mitral valve using clinically relevant cardiac imaging modalities.

## Methods

### Study population

All study procedures were approved by and in accordance with our laboratory’s Institutional Animal Care and Use Committee prior to initiating study activity. Sheep underwent preoperative CT scans and perioperative echo exams for use in a novel cardiac device study between June 2022 and February 2023. Fifty-five (n = 55) adult domestic sheep were to be scanned, examined, and included in the analysis.

### CT Scanning protocol

Sheep were anesthetized using methods described previously^21–24^. Briefly, peripheral intravenous access was established with a catheter placed in the external jugular vein, anesthetized with Propofol (1–2 mg/kg) intubated and connected to mechanical ventilation. Anesthesia was maintained with 1–3% isoflurane IN. Vital signs including ECG, pulse oximetry, temperature, and end tidal carbon dioxide were monitored throughout the procedure. Once prepared, animals were transferred to the CT suite. ECG-gated contrast enhanced CT scans were performed using a Siemens Somatom Dual Source Definition Flash Scanner. Standard computed tomography angiography (CTA) protocols were applied and scans performed with 128 × 0.6 collimation, 0.28 rotation time, 0.17 pitch, tube voltage of 100 kV, 1.0 mm slice thickness, and temporal resolution of 75 ms. Isovue-300 contrast was administered 1 mg/kg IV with a normal saline chaser bolus of 75 ml. Bolus tracking was placed at the aortic root at the level of the carina. Following acquisition, animals were removed from the scanner, weaned from anesthesia, and recovered to ambulatory.

### CT measurement protocol

**Figure 1:**
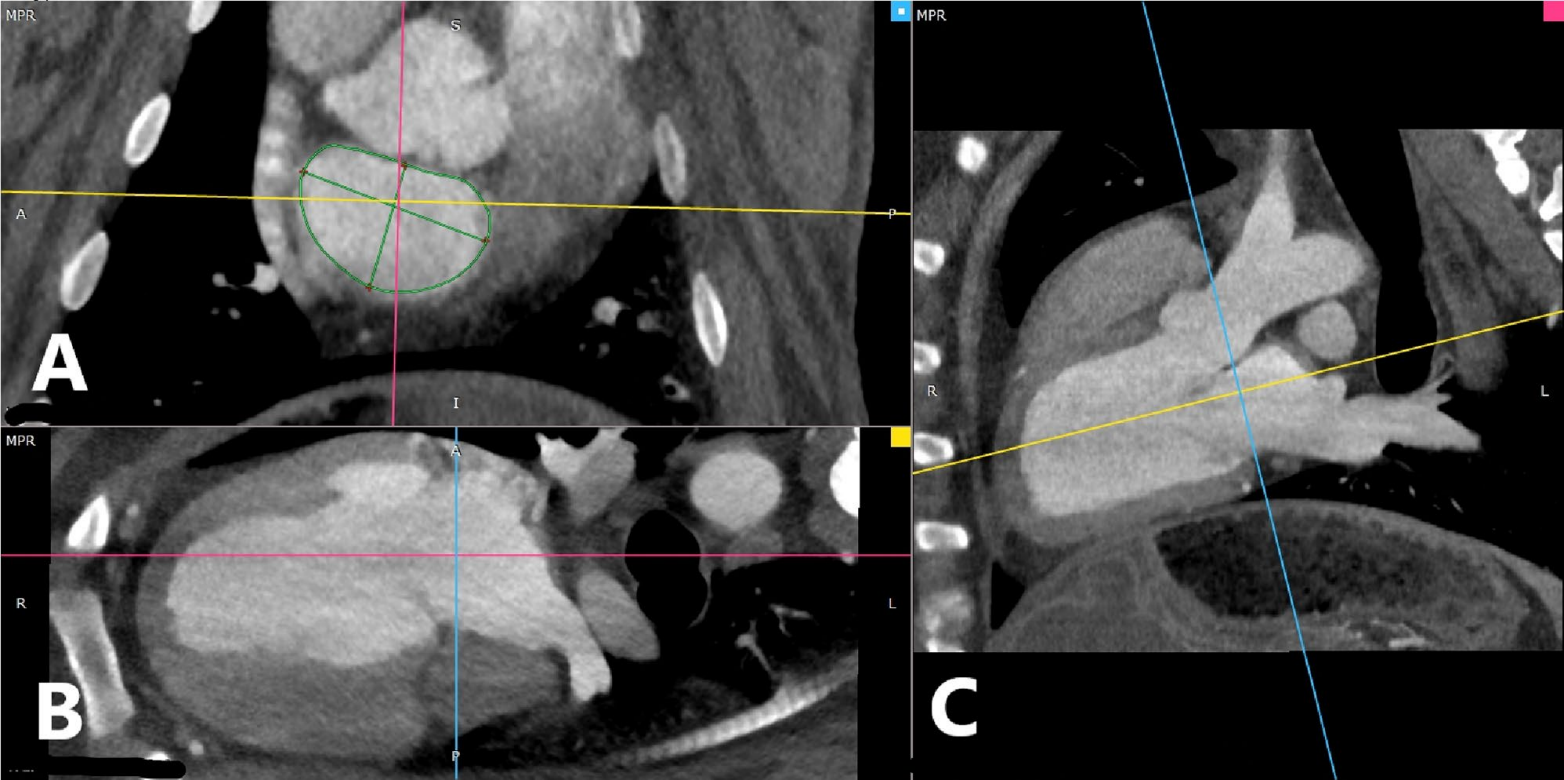
CT multiplanar reconstruction of the sheep heart as viewed from Sagittal (**A**), Axial (**B**) and Coronal (**C**) Windows. Note alignment of the sagittal plane (blue line) with the mitral valve annulus in the axial and coronal windows.

Mitral valve and associated left heart anatomic dimensions were measured during the diastolic phase of the cardiac cycle with RadiANT DICOM Viewer using its multiplanar reconstruction windowing package, as shown in Fig. 1. Multiplanar reconstruction sorts images captured by the camera into coronal, sagittal and axial windows. These views default to clinical patient positioning, with the patient lying prone in the scanner. While possible to scan sheep in dorsal recumbency, this requires positioning and balancing of the limbs due to the depth of their thorax. Additionally, in dorsal recumbency, the weight of the animal’s rumen can press on the inferior vena cava and restrict venous blood flow to the heart, resulting in sub-optimal images and concerns with anesthetic management. Lateral recumbency is far easier to stabilize the animal on the CT table, requiring no additional efforts to restrain the animal on the table, reducing anesthesia time.

**Figure 2:**
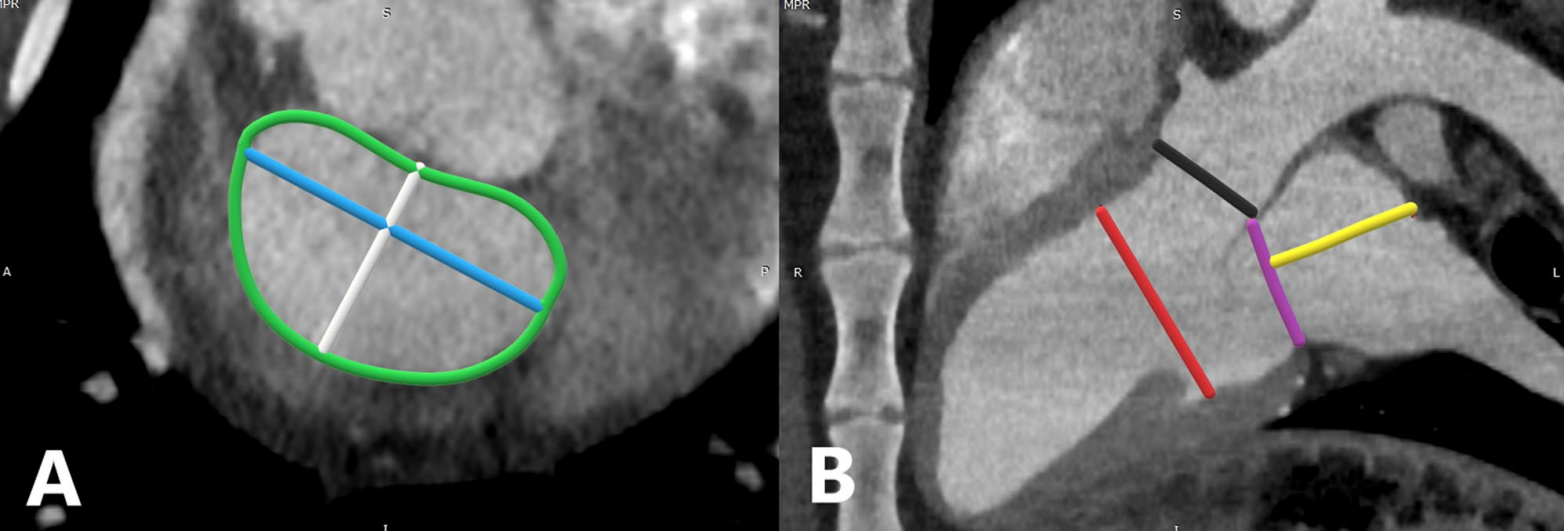
CT multiplanar reconstruction of the sheep mitral valve with annotated measurements. Image A presents the mitral valve as viewed in the sagittal window. Mitral valve dimensions presented are as follows: The mitral valve area and circumference is marked by the green line; The anterior–posterior leaflet dimension is marked by the blue line; The intercommissural dimension is marked by the white line. Image B presents the mitral valve as viewed in the coronal window. Dimensions are presented as follows: The left ventricle outflow tract is indicated by the black line; The left atrial height is indicated by the yellow line. The left ventricle inner diameter is marked by the red line; The purple line indicates the level of the mitral annulus for determination of left atrial height.

Segmentation and measurement of the mitral valve has been extensively described in the clinical literature^8–11,25^. Translating clinical CT reconstruction methods to the sheep model, we aligned the mitral valve in the axial, sagittal, and coronal planes at the level of the mitral annulus, as shown in Fig. 1. From the sagittal window, we measured valve area and circumference (MVA & C) inter-commissural diameter (IC), and septal-lateral aspect (AP), which is the distance between the anterior and posterior mitral valve leaflets. From the coronal window, we measured the diameter of the left ventricle outflow tract, mitral valve absolute diameter (an oblique anterior–posterior measurement), left atrial height as measured from the mitral valve annulus, and the inner diameter of the left ventricle at the level of the chordal attachment to the papillary muscles, as shown in Fig. 2.

### Echocardiography protocol and measurements

**Figure 3:**
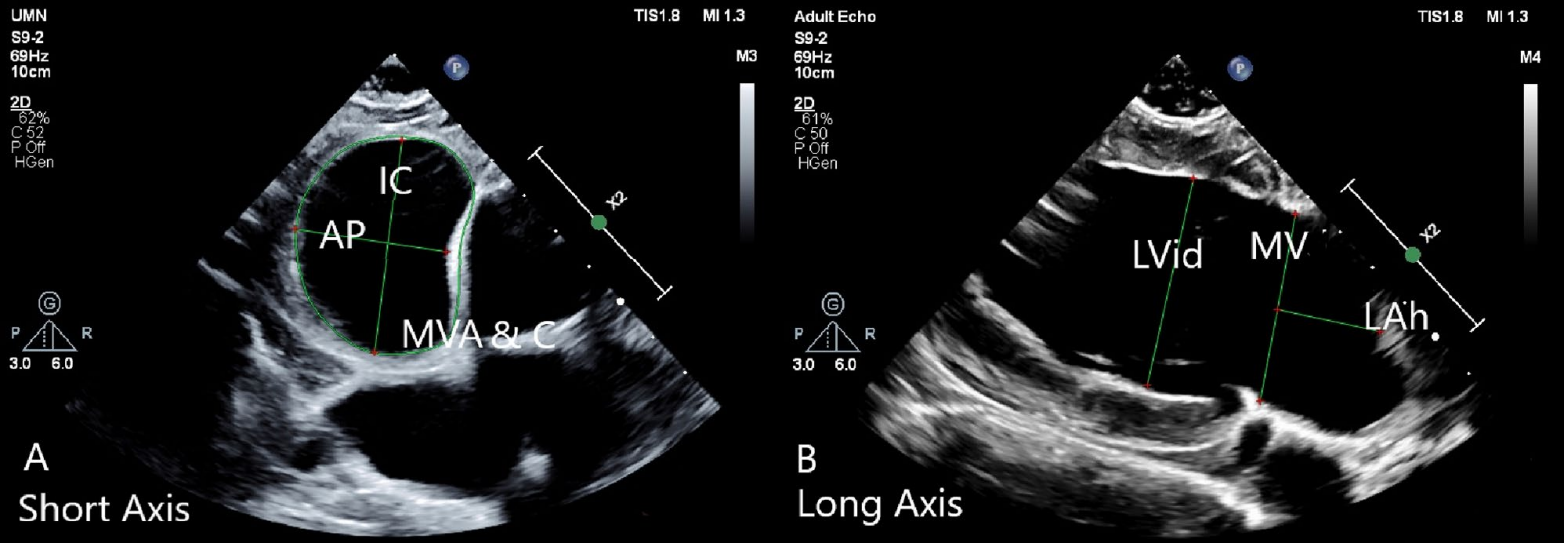
The mitral valve and left heart structures as viewed by echocardiography in short and long axis windows. Image (**A**) presents the short axis view of the mitral valve, with the inter-commissural (IC) and anterior–posterior (AP) dimensions, and mitral valve area and circumference (MVA & C) tracings indicated by green lines. Image (**B**) presents the long axis of the mitral valve, left ventricle and atrium. The left atrial height (LAh), mitral valve annular location (MV) and left ventricle inner diameter (LVid) are indicated by green lines.

Echos were collected perioperatively during a surgical procedure following CT scans, with animals fully anesthetized and prepared using methods described previously^21^. Exams were collected with a Philips Epiq CVx Cardiac Ultrasound machine with an S9-2 probe placed directly on the heart. Images of the mitral valve and left sided cardiac structures were collected in anatomical windows mirroring those acquired during CT scans. Following the echo exam, images were stored to the machine and later measured with RadiAnt DICOM reconstruction software. Measurements of the mitral valve and associated left heart structures was performed in equivalent orthogonal windows as prior CT scans as shown in Fig. 3.

### Dataset assembly and statistical analysis

All animal demographic information and measurements were performed in RadiAnt DICOM Viewer and recorded to Microsoft Excel worksheets. CT measurements were collected for all study animals prior to performing measurements derived from echo. CT and echo measurements were recorded in separate worksheets so as to not influence recording, then worksheets were combined upon completion of all data collected. Categorical variables were input as factors and reported as frequencies, while continuous variables were reported as (mean ± standard deviation). Two-sample t-tests were used to test for significance between dimensional measurements, with one and two-way ANOVA tests used to determine the effects of categorical variables on dimensional measurements. Linear Regression models and Pearson correlation coefficients were calculated to determine the relationships between age, weight and modality-related effects with Bland – Altman analysis performed to determine the mean difference in measurements between imaging modalities. All data analysis and statistics was performed using Rstudio.

### Ethical approval

All work performed in live animals was approved by the University of Minnesota’s Institutional Animal Care and Use Committee prior to study initiation. Authors confirm compliance with ARRIVE guidelines.

## Results

A total of fifty four (n = 54) adult domestic sheep, 18.3 ± 6.7 months of age, with 67% males (n = 36), weighing 82.7 ± 8.6 kg at the time of the initial CT scan were identified to fulfill the study cohort. One animal was excluded from the planned fifty-five animal study due to severe thoracic motion artifact during the CT scan. Three domestic sheep breeds were observed in the study population, Polypay Cross (n = 33), Friesian Cross (n = 9), and Suffolk Cross (n = 12) which are most commonly used in the laboratory. Average mitral annular dimensions measured by CT scan is presented in Table 1. The intercommissural diameter was determined to be 42.87 ± 2.84 mm; anterior–posterior leaflet dimension 28.89 ± 2.35 mm; circumference 121.11 ± 7.95 mm and area of 10.67 ± 1.39 cm^2^. The average left ventricle outflow tract diameter was 26.65 ± 2.62 mm; left ventricle inner diameter of 52.91 ± 3.28 mm and left atrial height 28.5 ± 3.51 mm. The average mitral annular dimensions measured by echo were an intercommissural diameter of 41.09 ± 2.91 mm; anterior–posterior leaflet dimension 28.61 ± 2.32 mm; circumference 119.5 ± 7.53 mm and area of 10.45 ± 1.29 cm^2^. The average left ventricle outflow tract diameter was 27.81 ± 2.79 mm; left ventricle inner diameter of 53.17 ± 4.89 mm and left atrial height 29.65 ± 4.18 mm. The only measurement that varied significantly between CT and Echo modalities was the intercommissural diameter (P = 0.0002).

**Table 1:** Study population measurements by imaging modality (n = 54)

Dimension	CT	Echo	P value
Mitral Intercommissural (mm)	42.87 ± 2.84	41.09 ± 2.91	0.00205*
Mitral Anterior–Posterior (mm)	28.89 ± 2.35	28.61 ± 2.32	0.352
Mitral Circumference (mm)	121.11 ± 7.95	119.5 ± 7.53	0.296
Mitral Area (cm^2^)	10.67 ± 1.39	10.45 ± 1.29	0.413
LVOT diameter (mm)	26.65 ± 2.62	27.81 ± 2.79	0.0548
Left Ventricle Inner Diameter (mm)	52.91 ± 3.28	53.17 ± 4.89	0.8901
Left Atrial Height (mm)	28.5 ± 3.51	29.65 ± 4.18	0.12151

Values expressed as (± s.d.).

*Significance expressed as P < 0.05 from two tailed t-test.

**Figure 4:**
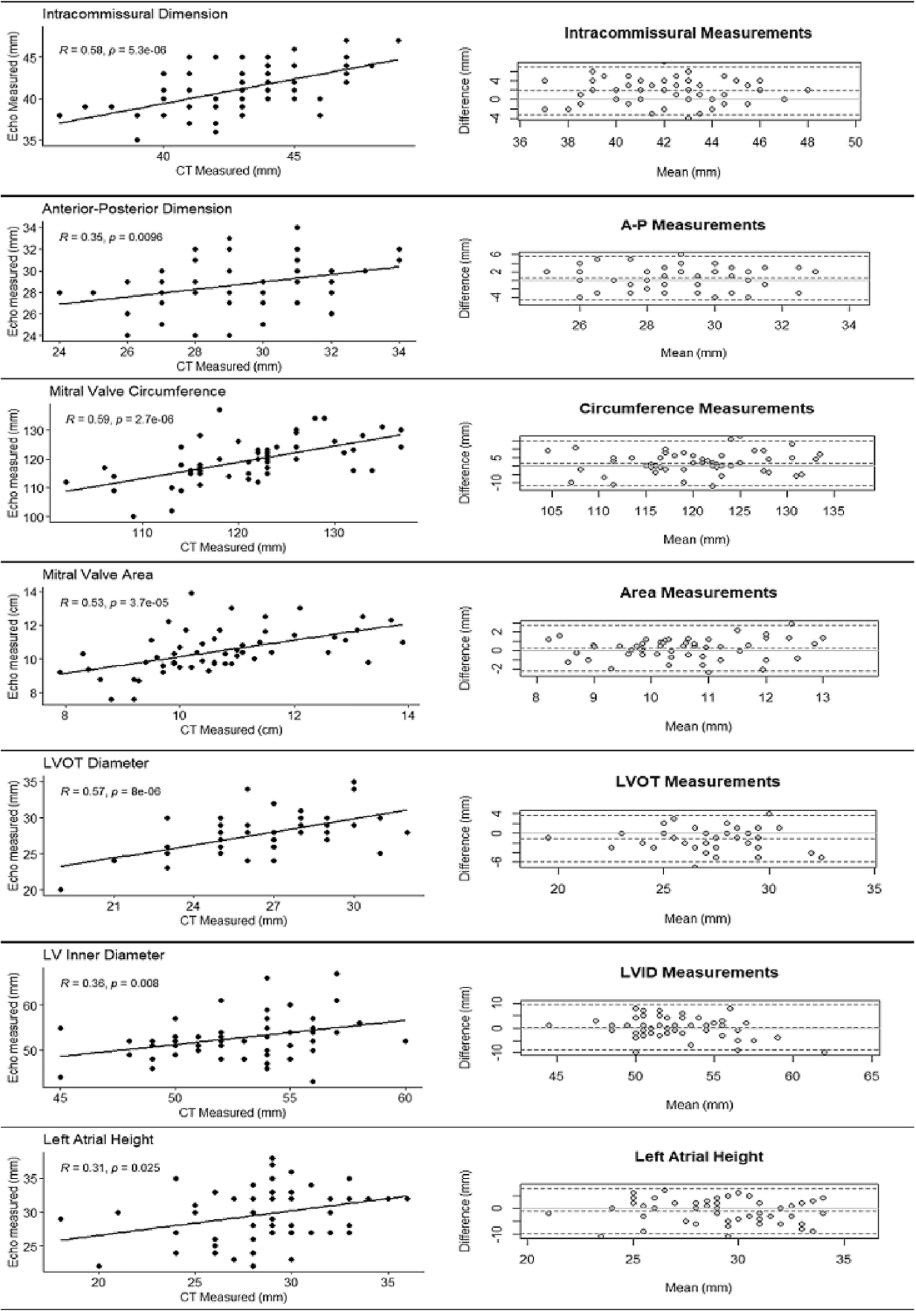
Regression analysis and Bland–Altman plots comparing measurements collected by CT and Echo. The column to the left displays the regression analysis with the column on the right the Bland Altman result from each dimensional measurement analyzed.

To compare dimensional measurements on the basis of imaging modality alone, a simple regression analysis for correlation and Bland–Altman analysis was performed for each dimensional measurement collected by both CT and Echo, as shown in Fig. 4. Moderate correlations were observed between CT and Echo derived measurements for Intercommissural (r = 0.58, P < 0.0001), Circumference (r = 0.59, P < 0.0001), Area (r = 0.53, P < 0.0001), and Left ventricle outflow tract (r = 0.57, P < 0.0001), while weaker correlations were observed for Anterior–Posterior (r = 0.35, p = 0.0096), Left ventricle inner diameter (r = 0.36, P = 0.0008) and Left atrial height (r = 0.31, P = 0.025).

**Figure 5:**
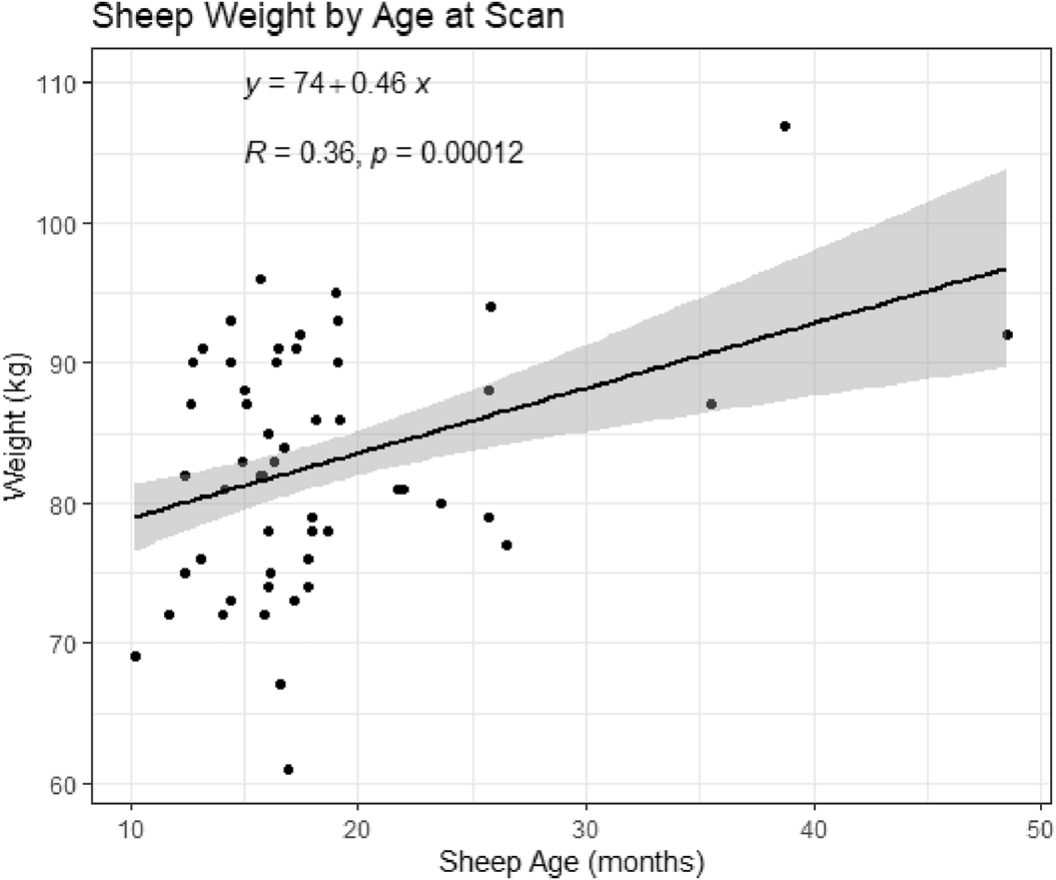
Regression analysis plotting sheep weight by age at the time of CT exam. The line of best fit is represented by the equation y = 74 + 0.46x, meaning that in the dataset, weight increased 0.46 kg for every one month of additional age.

Table 2 displays CT and Echo measurements analyzed by sheep breed. Breed did not appear to have any statistical effects on cardiac dimensional measurements in this study. Table 3 categorizes both CT and Echo derived measurements by animal sex, with the data displaying that there were no significant sex—determined effects on mitral dimensions observed in this study. We found a significant relationship between age and weight of the animals, (p < 0.0001, r = 0.36) with a 0.46 kg increase in weight for every one month increase in age, shown in Fig. 5. Linear regression analysis revealed that with the exception of left ventricle inner diameter and left atrial height, there was a statistically significant relationship between measured mitral valve dimensions and animal weight (p < 0.05). Age did not appear to have a significant effect on any measured mitral valve dimension when linear regression analysis was controlled by weight (p < 0.05).

**Table 2:** Measurements by breed and imaging modality

	CT				Echo			
	Friesian (9)	Polypay (33)	Suffolk (12)	P value	Friesian (9)	Polypay (33)	Suffolk (12)	P value
Sex	M:9; Fe:0	M:21: Fe:12	M:6; Fe:6		M:9; Fe:0	M:21: Fe:12	M:6; Fe:6	
Dimension								
Mitral Inter-Commissural (mm)	43.4 ± 2.7	42.6 ± 2.8	43.17 ± 3.3	0.683	41.33 ± 3.0	40.6 ± 2.9	42.2 ± 2.9	0.29
Mitral Septal-Lateral (mm)	29.2 ± 2.5	28.9 ± 2.5	29.2 ± 2.0	0.93	28.4 ± 2.4	28.9 ± 2.4	27.9 ± 2.0	0.442
Mitral Circumference (mm)	122.3 ± 7.5	121.0 ± 8.2	120.6 ± 8.2	0.875	119.4 ± 9.3	118.9 ± 7.1	121.08 ± 7.9	0.708
Mitral Area (cm^2^)	10.7 ± 1.4	10.7 ± 1.5	10.6 ± 1.3	0.983	10.37 ± 1.4	10.4 ± 1.3	10.6 ± 1.4	0.92
LVOT diameter (mm)	28.1 ± 2.6	25.8 ± 2.6	27.9 ± 1.9	0.079	28.2 ± 2.3	27.7 ± 3.0	27.83 ± 2.8	0.886
Left Ventricle Inner Diameter (mm)	53.0 ± 2.5	52.4 ± 3.4	54.3 ± 3.3	0.247	54.3 ± 6.8	51.8 ± 3.7	54.5 ± 5.74	0.148
Left Atrial Height (mm)	29.0 ± 2.8	28.9 ± 3.8	27.0 ± 2.8	0.248	29 ± 4.33	30.1 ± 4.1	29.0 ± 4.4	0.669

Values expressed as (± s.d.).

*M* male, *Fe* female.

*Significance expressed as P < 0.05 from two tailed t-test.

**Table 3:** Measurements by sex and imaging modality

Dimensions	CT			Echo		
	Male (36)	Female (18)	P value	Male (36)	Female (18)	P value
Mitral Inter-Commissural (mm)	42.7 ± 2.79	43.2 ± 2.98	0.524	40.9 ± 2.37	41.6 ± 3.81	0.413
Mitral Septal-Lateral (mm)	29.2 ± 2.27	28.7 ± 2.52	0.417	28.3 ± 2.14	29.2 ± 2.6	0.173
Mitral Circumference (mm)	121 ± 7.96	121 ± 8.14	0.83	119 ± 7.21	121 ± 8.21	0.383
Mitral Area (cm^2^)	10.7 ± 1.44	10.5 ± 1.31	0.59	10.3 ± 1.2	10.7 ± 1.44	0.255
LVOT diameter (mm)	26.6 ± 2.9	26.8 ± 2.01	0.17	27.5 ± 2.95	28.4 ± 2.38	0.244
Left Ventricle Inner Diameter (mm)	52.8 ± 3.05	53.1 ± 3.79	0.75	52.4 ± 4.38	53.6 ± 5.83	0.392
Left Atrial Height (mm)	28.4 ± 3.52	28.5 ± 3.60	0.89	29.4 ± 4.24	30.1 ± 4.14	0.617

(n =) is provided for sex; Values expressed as (± s.d.).

*Significance expressed as P < 0.05 from two tailed t-test.

## Discussion

The domestic sheep model has been used in long-term studies of novel cardiac valve replacement devices since the early 1990s^26–29^. Over the last three decades, there have been numerous publications describing the model’s use and expected outcomes for novel cardiac valve devices that have gone on to widespread clinical use and success. Surprisingly, little attention has been paid to characterizing the sheep model and its naive anatomy, especially using currently available imaging modalities like CT and echo, which are commonplace in the field of diagnostic cardiac clinical medicine. The closest an existing publication comes to the goals of the present study is the 2012 Halowell publication, which provides a comprehensive study of cardiac chamber indices and anatomical markers in sheep and goats by transthoracic echocardiography, but does not include valvular dimensional indices, data that is crucial to investigators using the sheep model in catheter-based cardiac valve replacement device studies^30^.

The gap in the scientific literature that we are presented with today could be an after-effect of the more recent increase in the development of and widespread clinical use of novel catheter-based cardiac devices. In previous decades, cardiac valvular dysfunction was predominantly treated with open-heart surgery facilitated by cardiopulmonary bypass (CPB). While some preoperative imaging in the form of CT or transthoracic echo would be performed to diagnose the cardiac disease or dysfunction, replacement of the diseased valve would be completed in a deeply anesthetized, stable patient, by a surgeon directly visualizing the insertion, seating and securement an appropriately sized device to match the patient’s anatomy, all while working in a relatively blood-free surgical field.

Patient-prosthesis mismatch (PPM) and its deleterious effect on the long term performance and use of heart valve replacement devices is a well-studied and documented adverse event in cardiac patients receiving heart valve replacement devices. Even under direct visualization in open heart surgery, PPM remains a primary concern for cardiac surgeons. This same effect is present in the catheter-based heart valve replacement field and is a shared concern between cardiac surgeons and interventional cardiologists performing the catheter-based valve replacement procedures. Unlike cardiac surgeons, interventionists do not have the advantage of direct visualization of the cardiac valve to be treated in order to select the appropriately sized device, rather, they must rely on preoperative and perioperative imaging of the patient to ensure a successful chronic treatment of the diseased valve. As a result, it is imperative that the most precise and accurate imaging methods are employed to ensure the success of a transcatheter valve replacement procedure.

Just as methods developed in research studies can be translated and applied to clinical use, the same methods developed in clinical use can be translated and applied to research studies. With this in mind, investigators in the catheter-based valve replacement field are requesting with increasingly frequency to implement and use pre- and perioperative imaging in their large animal research studies evaluating novel catheter-based valve replacement devices. Yet, little is known as to how to interpret these images in a large animal, especially in regards to anatomic sizing of the heart as imaged by CT and to an even lesser extent, general knowledge of the effects of sheep breed and sex on the normal indices of cardiac valvular dimensions in the model.

The objectives of the present study were to (1) investigate the feasibility and reproducibility of measuring mitral annular dimensions with clinically used CT reconstruction techniques, (2) characterize the dimensions of mitral valve annular and associated heart structure dimensions, and (3) compare CT-derived measurements to measurements collected from echocardiography exams performed in the same population. The CT-derived mitral annular and associated structure measurements presented in this study were completed using a clinical cardiac CTA protocol translated to the sheep model. All 54 sheep presented in this study were successfully anesthetized, scanned, and recovered without adverse event, then later used in a catheter-based mitral valve replacement surgery when the echo measurements presented in the study were obtained. Using the anesthesia protocol and scanning methods described, we propose that performing cardiac CT scans in sheep can be done reliably, quickly, and safely. Use of ECG gating and administration of short-acting propofol minimized effects of cardiac and thoracic motion artifact during the scans which resulted in high-fidelity and measurable images of the sheep’s mitral valve dimensions for preoperative planning.

Once enrolled in a surgical study, 2 dimensional echocardiography was performed perioperatively during the procedure. We sought to compare CT-derived measurements to echo-derived measurements of the same population in sheep. Echo with its elevated temporal resolution compared to CT, especially when applied to the epicardium of the heart, resulted in high-fidelity and detailed images of the beating heart in the same planes of measure obtained in CT scans. With this, echo-derived dimensions were measured and recorded in the same locations and viewing windows as CT measurements. Analyzing and displaying the CT-derived measurements in comparison to the echo-derived measurements, the only measurement with a statistically significant difference in values reported between CT and echo was the Intercommissural dimension, with an average length of 42.87 ± 2.84 mm on CT compared to 41.09 ± 2.91 mm on Echo (P = 0.00205). Investigators performing research at our laboratory sometimes specify a particular breed or sex of sheep to be enrolled in their cardiac valve replacement study, perceiving that one breed or sex is better suited to a certain anatomical dimension. Using one and two-way ANOVA tests we analyzed both CT and echo-derived measurements on the basis of animal breed and sex, we found no statistically significant causal or interaction effects on cardiac dimensions measured by either CT or Echo in this study. Publications in the clinical literature have shown that sex has significant effects on cardiac valve dimensions, but this is not reflected in our sheep data^33–35^. This could be due to the small sample size used in this study, or is indicative of a model-specific finding. Animal weight was shown to have a significant effect on mitral valve dimensions using linear regression analysis, while animal age at the time of the cardiac scans did not appear to have an effect on measured dimensions when controlled for by weight.

Reflecting on the significant difference observed in the intercommissural measurement between CT and echo, we are considering that there is another factor at work causing the variation in measurements between the two modalities. One which was not accounted for in our analysis, perhaps anesthesia time from when the animal was induced to the completion of the imaging procedure. CT scans typically take 15 min from the initial administration of anesthesia to completion of the scan, in comparison to a roughly 40 min for echo collected perioperatively. The hemodynamic effects of inhaled and injectable general anesthesia are well understood in clinical and veterinary medicine^31^. Perhaps this unexplored variable can be attributed to the observed variation in this cardiac dimension. Another possibility could be viewer error. Echo images are of higher temporal resolution than CT. This results in higher-fidelity imaging of small or thin structures. Unlike diseased mitral valve leaflets observed clinically, the mitral valve leaflets in healthy sheep are thin and fragile^32^. It is possible that the disparity between CT and echo measurements is a result of misinterpreting the true annular margin of the mitral valve annulus on the intercommissural dimension.

We found moderate agreement between CT and Echo-derived measurements of the same mitral annular and associated cardiac structures in the sheep using linear regression and Bland–Altman analysis. CT measurement of mitral structures tends to mildly over-estimate dimensional measurements, with the exception of left ventricle outflow tract diameter and left atrial height. CT measurements tended to under-estimate measurements of these dimensions in comparison to Echo. Once again, an unexplored variable such as anesthesia time, stroke volume, or atrial volume status when collected could explain the misalignment of the measurements, as the left atrial height is highly dependent on volume status of the heart, which is dependent on stroke volume, explained by Starling’s Law^36^. Ultimately, our hope is that this manuscript provides the first reproducible methods for assessment of the sheep model’s native cardiac valve anatomy using both CT and echo, and is a resource to future investigators using the model to advance novel device technology to clinical use.

### Limitations

There are several limitations to the present study. One is that the sheep used in this study have been purposely-bred for laboratory research use, and may not be indicative of the broader sheep population available to researchers in other areas. Sheep have been described by the animal vendors as either Polypay Cross, Friesian Cross, or Suffolk Cross, meaning that there has been some degree of cross-breeding acknowledged by the vendors. The extent of this is unknown as these animals are not genotyped, rather identified by the phenotype of their parents, spanning dozens of generations. Given the consistent similarities observed in cardiac measurements throughout this study, we speculate that the animals in the study share common ancestorial bloodlines. With this in mind, there could be significant differences in anatomical measurements between the cross-bred sheep used in this study compared to pure-bred bloodlines.

Lack of intra-observer measurements is another limitation of the study. The observer responsible for measuring the CT scans also performed measurements of the echo. Without a second observer to replicate these measurements, it’s entirely possible that measurements were collected in a manner which would differ from another in the field with the ability to measure and interpret both CT and echo-derived data.

Change in animal weight over time and its effect on cardiac dimensions was not evaluated in this study beyond a basic description, as the imaging time point between CT and Echo exams averaged 7 ± 4 days and during this time, and non-significant variations in animal weight were observed between the initial CT scans and the following perioperative echo exams. This is most likely due to the fact that sheep are housed in the same conditions and fed identical rations of feed per day. Had the duration between CT and echo been extended beyond 1 week, fluctuations in weight and its role in observed cardiac dimensions is an avenue of interest for future investigations.

We performed linear regression analysis comparing cardiac dimensional measurements to animal weight at the time of the CT scan. We found that weight had a statistically significant impact on mitral valve dimensions measured in this study, with the exception of the left ventricle diameter and left atrial height. As a result, in the adult sheep population, animal weight seems to be the most influential factor in determining and predicting mitral valve dimensions. Animal weight was shown to increase with age, but age did not appear to have a significant effect on cardiac dimensions when controlled for by weight. As our dataset was comprised of adult sheep, over the age of 9 months at the time of imaging, these results may not be representative of juvenile and adolescent sheep. Further study of cardiac growth curves in lambs are necessary to make predictions outside of the adult sheep population.

## Conclusion

In conclusion, this study has shown that application of clinical methods to cardiac CT imaging in sheep is a minimally invasive and consistent method to investigate mitral valve anatomy and associated structures in preparation for a catheter-based mitral valve replacement operation. To the authors’ knowledge, this is the first study of its kind investigating cardiac valve anatomical dimensions using clinically-relevant CT and echo imaging modalities. As in the clinical setting, we found that measurements derived from CT scans are comparable to those collected on cardiac Echo, and investigators in the field should expect highly correlated measurements between both imaging modalities in the sheep model. Breed and sex do not appear to elicit a significant effect on mitral valve dimensions or those of associated heart structures relevant in planning a TMVR operation. While the intercommissural dimension of the naive mitral valve appeared to significantly differ between CT and echo-derived measurements, the cause of this variation is unknown, but suspected to be a result of an unmeasured parameter in this study. This study will hopefully provide investigators in the transcatheter area the first available reference indices for mitral valve dimensions in sheep as measured by CT and echocardiography for use and comparison in future translational research studies of novel transcatheter valve replacement devices.

## Data Availability

Study data is available by reasonable request to the corresponding author.
